# Cellular Immunity Is Critical for Assessing COVID-19 Vaccine Effectiveness in Immunocompromised Individuals

**DOI:** 10.3389/fimmu.2022.880784

**Published:** 2022-05-26

**Authors:** Eustache Paramithiotis, Scott Sugden, Eszter Papp, Marie Bonhomme, Todd Chermak, Stephanie Y. Crawford, Stefanie Z. Demetriades, Gerson Galdos, Bruce L. Lambert, John Mattison, Thomas McDade, Stephane Pillet, Robert Murphy

**Affiliations:** ^1^ Research and Development, CellCarta, Montreal, QC, Canada; ^2^ Scientific Team, CellCarta, Montreal, QC, Canada; ^3^ Global Research and Development, CellCarta, Montreal, QC, Canada; ^4^ Vaccine Sciences Division, Pharmaceutical Product Development (PPD) Inc., Wilmington, NC, United States; ^5^ Regulatory and Government Affairs, CellCarta, Montreal, QC, Canada; ^6^ Department of Pharmacy Systems, Outcomes and Policy, University of Illinois Chicago, Chicago, IL, United States; ^7^ Center for Communication and Health, Northwestern University, Evanston, IL, United States; ^8^ Robert J. Havey, MD Institute for Global Health, Northwestern University, Chicago, IL, United States; ^9^ Health Information, Kaiser Permanente, Pasadena, CA, United States; ^10^ Health Technology Advisory Board, Arsenal Capital, New York, NY, United States; ^11^ Department of Anthropology, Northwestern University, Evanston, IL, United States; ^12^ McGill University Health Center, Montreal, QC, Canada

**Keywords:** cellular immunity, immunocompromised, vaccine, COVID-19, efficacy

## Abstract

COVID-19 vaccine clinical development was conducted with unprecedented speed. Immunity measurements were concentrated on the antibody response which left significant gaps in our understanding how robust and long-lasting immune protection develops. Better understanding the cellular immune response will fill those gaps, especially in the elderly and immunocompromised populations which not only have the highest risk for severe infection, but also frequently have inadequate antibody responses. Although cellular immunity measurements are more logistically complex to conduct for clinical trials compared to antibody measurements, the feasibility and benefit of doing them in clinical trials has been demonstrated and so should be more widely adopted. Adding significant cellular response metrics will provide a deeper understanding of the overall immune response to COVID-19 vaccination, which will significantly inform vaccination strategies for the most vulnerable populations. Better monitoring of overall immunity will also substantially benefit other vaccine development efforts, and indeed any therapies that involve the immune system as part of the therapeutic strategy.

## Introduction

The historically fast delivery of COVID-19 vaccines has already saved upwards of a million lives globally (1). Vaccine clinical development, however, focused heavily on the antibody response while largely neglecting the cellular response. While this narrower focus may have accelerated deployment of the vaccines, it also left significant gaps in our understanding of how robust and long-lasting protection develops. A deeper understanding of cellular immunity will help fill those gaps, and this information will be important in formulating vaccination strategies for situations where the antibody response alone is insufficient when developing strategies to combat new viral variants, addressing questions about mix-and-match vaccination, booster vaccinations, and optimizing vaccination strategies for individuals that are elderly or immunocompromised by diseases or therapy.

In this paper we argue that COVID-19 vaccination trials need to include more extensive assessment of cellular immunity, especially for immune compromised individuals. The experimental and logistical technologies necessary to effectively conduct more complete assessments of cellular immunity already exist and are being used in other kinds of clinical development. Immunocompromised individuals are at higher risk for contracting severe COVID-19 disease and are more likely to insufficiently respond to the existing vaccination regimens. They will be the biggest beneficiaries of introducing more comprehensive immune evaluation of vaccine responses, and therefore the place to start.

The arguments presented here in favor of more comprehensive cellular immune response assessments have been made in a COVID-19 vaccination specific context. However, many of the knowledge gaps identified below are not unique to COVID-19. They also apply to other vaccine development efforts, and indeed to any therapies that recruit the immune system as part of the therapeutic strategy. Therefore, other significant populations beyond those affected by SARS-CoV-2 infection, such as type-2 diabetes, obesity, HIV, or other chronic infections will also benefit from more comprehensive assessments of cellular immunity.

## Evaluating Immunity in Immunocompromised Subjects

Immune responses are highly coordinated, involving multiple cell types and soluble factors, and result in both antibody-derived and cellular immunity (2, 3). These two types of immunity are complementary and interdependent. Antibodies prevent infection by blocking entry of pathogens into target cells, whereas cellular immunity clears infection by removing infected cells. The generation of high-affinity immunoglobulin G (IgG) antibody responses depend on the presence of cellular immunity, particularly CD4^+^ T helper cells. The development of cellular immunity is in turn accelerated by opsonization, which is the absorption and processing of antibody-pathogen complexes by immune cells. Recognizing this integration of cellular and humoral immunity, the European Roadmap for Vaccine Development called for immunogenicity testing to include both antibody and cellular immunity functions (4).

In healthy individuals, antibody measurements have been used as surrogates for overall responsiveness to vaccination and correlate well with measures of cellular immunity (5, 6). However, the kinetics of antibody and cellular responses differ, as shown by the differential waning of antibody levels compared to cellular immune responses following vaccination against SAR-CoV-2 (7–13). T cell responses may thus not be accurately predictable by inference from antibody data alone. Therefore, for a more complete understanding of immune protection, measures of both antibody-based as well as cellular immunity are required. In immunocompromised populations, where antibody measurements alone provide a much less complete assessment of the response to vaccination, adding measures of cellular immunity is urgently needed. The characteristics of the largest of the immunocompromised subgroups in the context of SARS-CoV-2 infection and vaccination are discussed below.

### Inborn Errors of Immunity

Most immune deficiencies are acquired, developing because of chronic disease, or immune suppressant therapies. A small proportion (~4%) have genetic origins, including genetic susceptibility to infectious triggers of autoimmune diseases. The vast majority of these inborn errors of immunity result in defects in antibody production (14). An extreme of this diverse group are agammaglobulinemic persons, who are otherwise healthy but lack all antibody production. Nonetheless, they can successfully mount T cell-based immune responses against many microbial infections (15).

Abnormalities in antibody production make standard measures of antibody titer difficult to interpret and of limited usefulness in this population. For example, analysis of antibody and cellular immunity responses after a single Pfizer-BioNTech SARS-CoV-2 vaccination in patients with multiple kinds of inborn errors of immunity revealed highly variable antibody responses that did not reach protective levels in a large fraction of subjects (16). In contrast, T cell responses were comparable between those with inborn immune errors, the healthy vaccinated, and healthy convalescent control populations. The best predictor of immune responsiveness was the cellular immune status prior to vaccination (16). Deficient antibody production after vaccination in subjects with primary antibody deficiencies was also reported by Selinas et al. (17), who further described atypical memory B cells but mostly intact T cell responses in these subjects. Increased memory B cell frequency was also associated with response to vaccination (18). Furthermore, a study of subjects with B cell compartments compromised either by inherited immune deficiencies, blood cancers, or therapy determined that the number of naïve B cells available was an independent predictor of successful vaccination (19). Taken together these findings suggest that exclusive reliance on antibody testing would likely underestimate vaccine responsiveness for a large segment of individuals with inherited immune deficiencies. A better characterization of the deficient B cells would improve our understanding of the impact of those deficiencies on vaccine provided protection while helping to develop new vaccine candidates for particularly susceptible populations.

### Cancer

Individuals with cancer generally have suppressed immune function as a result of the disease (20–24) and of the treatment (25–27). Unsurprisingly, cancer patients with SARS-CoV-2 infections have substantially reduced antibody responses, the vast majority of which did not generate sufficient humoral immunity (27–33).

Persons with hematologic cancers, however, that maintained CD8 (cytotoxic) T cell numbers above a defined threshold had better survival rates than those who could not maintain such levels (33). Thus, simply counting the CD8 T cells may identify most of the hematological cancer patients with the highest risk of severe COVID-19 infection and death. Including these measures as part of regular assessment may allow for preventative measures that improve survival. The authors concluded that boosting T cell immunity in cancer patients would substantially aid their ability to weather an infection (33). This interpretation was further supported by other studies of SARS-CoV-2 vaccine responses in patients with cancer (29, 30, 34). Most patients with impaired antibody responses generated protective levels of cellular immunity, suggesting that cellular immunity may be able to overcome the absence of an antibody response and clear the infection. For patients immunocompromised by cancer, relying on antibody testing alone will be unable to distinguish those who can respond to vaccination from those who cannot. Measurements of cellular immunity can provide those distinctions and help evaluate novel strategies to boost immunity in cancer patients.

### Immune Suppressive Treatment

Intentional or collateral immunosuppression may result from therapeutic treatments targeting a wide variety of conditions. Immunosuppressive therapies are a staple for transplant recipients and for those with autoimmune morbidities. Among all immune supressed individuals, transplant recipients produced the weakest response to vaccination (35, 36), consistent with the severe immune suppression intentionally induced to prevent transplant rejection. Researchers observed extremely low antibody responses following both SARS-CoV-2 infection (35) and vaccination (36–38). Cellular immune responses, however, impacted outcome severity (35), again illustrating the importance of cellular immunity in resolving SARS-CoV-2 infection in the immunocompromised. Testing for cellular immune responses in transplant recipients may enable clinicians to identify which subjects are at higher risk of not controlling an infection.

Many people receive immunosuppressive treatment for a large variety of autoimmune conditions. These include arthritis, inflammatory bowel disease, and multiple sclerosis, among many others (39, 40). In addition, systemic inflammation is known to suppress immune function (41, 42) and is associated with the development of heart disease, obesity, metabolic syndrome, and type 2 diabetes (41, 42). In total, there are potentially millions of people living with varying degrees of immune deficiency caused by these conditions, and these immune deficiencies may significantly affect the ability to produce humoral or cellular immune responses to vaccination. Despite being high risk for severe disease, these groups were underrepresented in first-generation COVID-19 vaccine trials. Conducting more comprehensive assessments of immune responsiveness that include assessments of cellular immunity would enable a more complete understanding of immune status and of the interventions necessary to establish immunity in these individuals.

For example, a study of COVID-19 vaccinated patients with psoriasis receiving immunosuppressant drugs (43) demonstrated variable, but overall poorer antibody responses compared to a control population. Their cellular immune responses, however, were at least as robust as the responses of the controls (43). This differential response to vaccination would not have been observed if only antibody titer measurements were used. Additionally, some treatment types appeared comparatively more immune sparing than others, which may become an important consideration when selecting therapeutic options. Thus, manufacturers of immune suppressants and treating physicians would benefit from a more complete evaluation of the impact immunosuppressant therapies have on SARS-CoV-2 vaccine efficacy. Including cellular immunity measurements along with antibody titer assessments will begin to fill those gaps.

### Aging

Aging in healthy individuals is independently associated with a decline of immune responsiveness due to a reduction in the total number of immune cells available as well as a specific reduction in the ability to respond to novel antigens (44–46). Even healthy elderly have immune systems that differ significantly from younger adults (44–48) and typically require higher vaccine doses to elicit a sufficient immune response (44, 49–51). Chronic diseases, infections, and other underlying health conditions can combine with the effects of aging to further weaken the immune systems of elderly individuals. As a result, there is an increased vulnerability to external stressors, including infections (52) and a significantly higher risk of vaccine failure (53).

The effects of aging are not uniform. Recent studies define approximately half the over-55 population as immunologically frail (54, 55). Greater immunological frailty was associated with a poorer response to vaccination (55). Subsequent studies have further supported the association of frailty with poor immune responses (56), and diminished vaccination efficacy (56–58). In SARS-CoV-2 infection, frailty correlated with severe disease and mortality more than chronological age (59–61). However, the SARS-CoV-2 vaccine trials to date have excluded volunteers with severe comorbidities and conditions impacting immune functioning (62–64), even in vaccine efficacy studies in the elderly (49). Thus, vaccine efficacy in the frail elderly remains unknown. The elderly share with the other immunocompromised populations described here a significant unmet need for comprehensive evaluation of their immune systems to inform strategies to risk stratify patients and to increase vaccine response in those identified as high risk.

### Immunocompromised Population Size

There are potentially many millions of individuals with at least partial immune deficiency. Approximately 4% of US residents have inherited or acquired immune deficiencies (53) and approximately 16% of the US population is over 65 years old. About 2.5% of the US population lived with cancer in 2020 (65), and millions receive immune suppressants for cancer or autoimmune diseases. Even with a significant overlap between these populations, millions of Americans are affected, rendering them at least partially immunocompromised. In persons younger than 65, at least several million may also be indirectly immunocompromised due to obesity, diabetes, and heart disease. Comparable populations are also expected in other developed societies. The size of these populations and the lack of understanding of cellular immune response to vaccination, emphasizes the urgency of the unmet need.

Immunocompromised persons are a significant segment of the population. They are also the most at risk for severe SARS-CoV-2 infection. By excluding these persons from clinical trials, particularly larger-scale phase 2 and 3 trials represent a potential source of observational bias and has led to large gaps in understanding of the true efficacy of vaccination. By including T cell evaluations in clinical development programs, immunocompromised persons no longer need to be excluded for fear of confounding study data. To the contrary, optimal vaccination strategies can be designed specifically to target these groups.

## COVID-19 Specific Issues

As outlined above, individuals can become immunocompromised by age, disease, or chronic treatment. This reduces their ability to mount robust immune responses to challenges, including vaccination. Better understanding both the cellular and antibody responses in these individuals will inform more effective vaccination strategies. In addition to this generally applicable observation, several COVID-19 specific issues have emerged which further underscore the need for a greater understanding of the overall vaccine-induced immune response.

### Novel Viral Variants

The generation of new viral variants of concern has been discussed extensively elsewhere (66). Viral variants have been repeatedly shown to reduce the effectiveness of neutralizing antibodies developed through vaccination with the prototype strain (67). The corresponding T cell responses, however, remained robust against the variants (67, 68), largely because T cell responses were less sensitive to the single amino acid mutations that define emerging variants. Moreover, T cells have been identified that target sections of the SARS-CoV-2 proteins which cannot mutate without compromising viral fitness (69–71). These T cells, if induced by vaccination or infection by the ancestral strain of SARS-CoV-2, will provide cross-protective benefits against many mutant variants. Indeed, similar cross-protection has been demonstrated with several other viral infections including influenza (72–74), as well as other anti-viral vaccines (75, 76), re-enforcing that this is by no means a COVID-19 specific effect.

T cell response to vaccination may provide the missing link between experimental testing and what is observed empirically in the real world. As mentioned above, highly infectious variants exhibit mutations that were demonstrated to provide immune escape from neutralizing antibodies induced by either infection with the ancestral strain or vaccination (77). Therefore, quantitative, and qualitative analysis of T cell responses induced by the different vaccine candidates should be considered when evaluating the utility of any vaccination strategy or regime, including the need of a third dose booster shoot.

By focusing on antibody testing in vaccine clinical trials, these robust cross-reactive T cell populations remain understudied, limiting our true understanding of vaccine efficacy. As an example, using flow cytometry several recent independent studies have confirmed that the vast majority (>90%) of the T cell response induced by two doses of mRNA vaccines were directed against conserved, “mutation-resistant” sections of the virus (78, 79). When single mutations do occur in these regions, they had limited-to-no impact on T cell activation. However, these studies were limited in size relative to large phase-III clinical trials and lack the regulatory rigor required to submit for vaccine licensing. T cell evaluations of this type must be taken from the academic realm and applied into the world of vaccine clinical trials.

### Long COVID

Long COVID-19 is defined as sequelae that extend beyond four weeks after initial infection (80). Statistically significant risk factors included age, with the ≥50 age group having the greatest risk, and number of pre-existing medical conditions, in particular hypertension, obesity, psychiatric or immunosuppressive conditions (81). Chronic fatigue was the most frequently reported symptom following recovery from acute infection (82–85). Other frequently reported symptoms included dyspnea, cardiovascular abnormalities, cognitive impairment (“brain fog”), smell and taste dysfunction, and other less common symptoms (82, 86–93).

The mechanisms leading to long COVID-19 are not fully understood but may involve deficits in cellular immune function. A study that showed no difference in severity of initial infection between individuals with and without post-acute infection sequelae identified lower and more rapidly waning specific T cell responses in the latter population (94). In subjects experiencing neurological post-acute sequelae, no correlation between anti-SARS-CoV-2 T cell responses and IgG production could be found at 155-315 days post-infection, indicating that antibody responses cannot serve as a surrogate indicator of cell-mediated immunity in this group (95). This study also described aberrant T cell memory responses to vaccination in subjects with neurological long COVID-19 (95). The finding that some people experiencing long COVID symptoms notice an improvement after vaccination, and others a worsening of their symptoms supports the potential role of a heterogeneous immune response in the pathogenesis of long COVID (96). Taken together these observations underscore the need for independent monitoring of these two arms of the immune system when following long COVID-19 patients. While our understanding of long COVID-19 is still emerging, currently it appears that the elderly and immunocompromised individuals are most at risk, and that the cellular immune response may be disproportionally affected. Thus, like the other immunocompromised groups described above, long COVID-19 sufferers would also benefit from a comprehensive evaluation of their immune responses to vaccination.

### ‘Mix-and-Match’ Vaccination Strategies

Concurrent with the rise of more infectious viral variants, recommendations in favor of inoculations with more than one type of SARS-CoV-2 vaccine, and clinical studies evaluating the effectiveness of vaccine mixing and matching have been presented (97, 98), and many governments are considering mix-and-matched 3^rd^ boosters shots for deployment amongst their populations. The argument for comprehensive immune monitoring applies when considering mix-and-matching vaccination are similar to the arguments presented for viral variants or immunocompromised persons. Different SARS-CoV-2 vaccines have been shown to induce T cell responses that can differ in their magnitude, as well as the composition of the T cell response (99). T cell immunity may not be qualitatively the same in all mix-and-match vaccination situations. It cannot be predicted how different vaccines will complement or conflict with each other when used in any given mix-and-match vaccination strategy, nor can it be taken for granted that the quality or quantity of a T cell immune response can be accurately inferred by evaluating antibody responses alone. Therefore, when assessing which mix-and-match vaccination strategies are the best choice against all viral variants, T cell immune monitoring must be included in these clinical trials. This is particularly important when considering immunocompromised groups of persons, as discussed above.

Such an update to our research paradigm will lead to better-informed health policy decisions and establish more robust clinical decision criteria not only for this current pandemic, but for other contemporary vaccination campaigns, as well as future pandemic diseases. These new vaccination strategies could be tailored at the population level to optimize vaccine choices for particular age groups and/or disease states. At the most extreme, vaccination strategies could be personalized based on particular patient idiosyncrasies.

## Measurements of Cellular Immunity

The generally accepted gold standard for vaccine clinical trials is measurement of antibody titre and neutralizing activity. These tests can be done to scale with readily obtainable blood plasma or sera and have been an adequate surrogate of the overall immune response in healthy populations. In contrast, measurements of cellular immunity require living peripheral blood mononuclear cell (PBMC) samples which have more complex handling requirements. These logistical deterrents were not insurmountable as cellular immunity measurements are commonplace in a variety of clinical trials, including examples in CAR-T (100), immunomodulatory oncology treatments (43, 101), HIV (102), novel flu vaccination (103, 104), minimal residual disease (MRD) assessment (105, 106) and others (107, 108). However, it should be noted that cellular immunity assays are typically limited to early stage and smaller clinical trials.

In addition, novel techniques to measure cellular immunity from large groups of participants in non-clinical settings have been implemented (109, 110). These methods can be readily adapted to COVID-19. While research in non-clinical settings may not be able to match the depth of immunophenotyping that is possible in the lab it can provide complementary breadth by reaching more participants in order to laying the groundwork for more focused large scale clinical investigations. Basic science helps identify key biologic markers involved in processes relevant to disease progression and the host response. Applied science then explores those biomarkers within specific clinical use cases. As clinical understanding matures to the point where the key biomarkers in a particular process become apparent, the next phase identifies a restricted set of biomarkers that are most useful, scalable, and inexpensive. A deeper investment in a comprehensive immunologic profiling now will be critical to defining the inexpensive and scalable biometric assays that will become widely used in clinical care later.

ELISpot (enzyme-linked immune absorbent spot) and flow cytometry are currently the two main technologies used to conduct cellular immunity measurements in vaccine trials (111, 112). Both measure the frequency of cytokine-secreting PBMCs after antigen-specific activation in culture and provide an indication of response magnitude. However, ELISpot does not characterize the responding cells, whereas flow cytometry can identify the type of cell responding to activation. This additional characterization is necessary for a full understanding of the cellular responses induced by vaccination. This is important because responses can be derived from naïve, memory, regulatory, or effector cell types, all of which have different roles in establishing and maintaining long-term immunity. Understanding what type of cells are involved allows for a far more robust characterization of the immune response, which may be particularly important to know in immunocompromised individuals.

Although flow cytometry generates more information per sample, ELISpot is still dominant in vaccine trials, primarily due to cost considerations and perceptions of better standardization for regulatory submissions. Best practice guidelines have been published to guide flow cytometry data evaluation in a standardized manner (109, 113–117). Guidelines for general method validation (114, 118–120), as well as recommendations for specific applications are widely available (121, 122). Furthermore, additional, unsupervised flow cytometry data evaluation methods have been developed in recent years (123–125) as the number of parameters that can be simultaneously interrogated by flow cytometry has increased (125–129). Therefore, there are few technical limitations preventing regular deployment of multiparametric flow cytometry for an in-depth and standardized characterization of cellular immunity in vaccine clinical trials.

ELISpot tests can be run with fewer cells and fewer reagents than multi-parametric flow cytometry, although ELISpot tests typically require significantly more culture time. However, the principal fixed costs of cellular immunity measurements are derived from the need for living cells, and thus the establishment and execution of the highly qualified sample procurement, preparation, and transport protocols. The costs associated with these activities are the same, regardless of whether ELISpot or flow cytometry would be used. We argue that multi-parametric assessment is not only a better return on that investment, but is essential to the evaluation of vaccine development programs. This is especially true in test populations that lack previous cellular response characterization. This logic would apply equally to the current COVID-19 pandemic as to future vaccination programs. Costs can also be reduced by reserving comprehensive immune response analysis of both antibody and cellular immunity measurements to specific subpopulations, enabling the inclusion of immune deficient and frail elderly subjects into the large vaccine trials. Neglecting to include detailed cellular immunity evaluations in clinical trials now will likely result in collateral costs to public health and healthcare systems that far outweigh the price of these tests.

## Conclusion

In summary, we advocate for much more holistic assessment of immune status in research cohorts as a prerequisite for advancing both population-based and personalized vaccination strategies. We believe that boarder research testing, including T cell evaluations, will lead to better-informed anti-COVID-19 vaccination strategies which are effective, efficient, and inexpensive. This is particularly important in immunocompromised populations, including those suffering from long COVID-19, where antibody production may be absent or aberrant, and therefore vaccine protection is largely dependent on the T cell-mediated arm of the immune system.

The paradigm surrounding immune monitoring for infectious diseases and their corresponding vaccines needs an update. Regulatory bodies, vaccine manufactures, manufactures of immune-suppressive therapeutics, physicians, and others must shift their thinking away from an antibody-only approach towards a more holistic approach including the monitoring of T cell mediated immunity. There is an urgent need to reframe our research paradigm immediately while recruitment of individuals for prospective vaccination studies is still relatively easy. The technologies and logistic channels to perform T cell testing are well established and readily available. These assessments are essential, the cost of not performing them will be paid in financial and operative stress to our healthcare systems, as well as in human morbidity and mortality, particularly amongst immunocompromised groups.

## Data Availability Statement

The original contributions presented in the study are included in the article/supplementary material. Further inquiries can be directed to the corresponding author.

## Author Contributions

All authors listed have made a substantial, direct, and intellectual contribution to the work, and approved it for publication.

## Conflict of Interest

EuP, SS, EsP, and TC are employed by CellCarta Biosciences Inc., MB is employed by PPD, Inc., JM is employed by Arsenal Capital.

The remaining authors declare that the research was conducted in the absence of any commercial or financial relationships that could be construed as a potential conflict of interest.

## Publisher’s Note

All claims expressed in this article are solely those of the authors and do not necessarily represent those of their affiliated organizations, or those of the publisher, the editors and the reviewers. Any product that may be evaluated in this article, or claim that may be made by its manufacturer, is not guaranteed or endorsed by the publisher.
